# Activation of the AMPK-ULK1 pathway plays an important role in autophagy during prion infection

**DOI:** 10.1038/srep14728

**Published:** 2015-10-01

**Authors:** Xue-Yu Fan, Chan Tian, Hui Wang, Yin Xu, Ke Ren, Bao-Yun Zhang, Chen Gao, Qi Shi, Ge Meng, Lu-Bin Zhang, Yang-Jing Zhao, Qi-Xiang Shao, Xiao-Ping Dong

**Affiliations:** 1 Department of Immunology, and Jiangsu Key Laboratory of Medical Science and Laboratory Medicine, School of Medicine, Jiangsu University, Zhenjiang 212013, Jiangsu, China; 2 State Key Laboratory for Infectious Disease Prevention and Control, Collaborative Innovation Center for Diagnosis and Treatment of Infectious Diseases (Zhejiang University, Hangzhou 310003), National Institute for Viral Disease Control and Prevention, Chinese Center for Disease Control and Prevention, Chang-Bai Rd 155, Beijing 102206, China; 3 Chinese Academy of Sciences Key Laboratory of Pathogenic Microbiology and Immunology, Institute of Microbiology, Chinese Academy of Sciences, Beijing 100101, China

## Abstract

AMPK is a serine/threonine protein kinase that acts as a positive regulator of autophagy, by phosphorylating ULK1 at specific sites. A previous study demonstrated activation of the macroautophagic system in scrapie-infected experimental rodents and in certain human prion diseases, in which the essential negative regulator mTOR is severely inhibited. In this study, AMPK and ULK1 in the brains of hamsters infected with scrapie strain 263 K and in the scrapie-infected cell line SMB-S15 were analysed. The results showed an up-regulated trend of AMPK and AMPK-Thr172, ULK1 and ULK1-Ser555. Increases in brain AMPK and ULK1 occurred at an early stage of agent 263 K infection. The level of phosphorylated ULK1-Ser757 decreased during mid-infection and was only negligibly present at the terminal stage, a pattern that suggested a close relationship of the phosphorylated protein with altered endogenous mTOR. In addition, the level of LKB1 associated with AMPK activation was selectively increased at the early and middle stages of infection. Knockdown of endogenous ULK1 in SMB-S15 cells inhibited LC3 lipidation. These results showed that, in addition to the abolishment of the mTOR regulatory pathway, activation of the AMPK-ULK1 pathway during prion infection contributes to autophagy activation in prion-infected brain tissues.

Prion diseases, also known as transmissible spongiform encephalopathies (TSEs), are a group of fatal neurodegenerative disorders that include Creutzfeldt–Jakob disease (CJD) in humans and bovine spongiform encephalopathy (BSE) and scrapie in animals^1^. It is widely accepted that Prion diseases are caused by unconventional infectious agents termed “prions” that lack nucleic acids. During infection, a host-encoded glycoprotein PrP^C^ becomes the misfolded conformer PrP^Sc^, which accumulates mainly in the central nervous system^2^. Spongiform change, neuronal loss and gliosis (astro- and microglia) are the neuropathological hallmarks of prion diseases.

During autophagy, cytoplasmic material, organelles and misfolded proteins are degraded and digested within lysosomes^3^. Autophagy is activated by various environmental stresses and is rapidly up-regulated when cells need to generate intracellular nutrients and energy, for example, during starvation, growth factor withdrawal, or high bioenergetic demands. More than 30 different autophagy-related genes (ATG) have been identified. Autophagy is also associated with a range of human pathophysiologies, including cancer, neurodegeneration and cardiomyopathies^4^. Numerous Studies have provided the evidences that autophagy involves in the clearance of misfolded proteins and subsequently neuron damage during the pathogenesis of neurodegenerative disease^5^,^6^. Among the autophagic pathways identified in cells are macroautophagy, microautophagy and chaperone-mediated autophagy^7^,^8^,^9^. The mammalian target of rapamycin (mTOR)^10^ is a negative regulator of autophagy while the BECN1-PIK3C3/Vps34 (vacuolar sorting protein34)-Vps15 (vacuolar sorting protein15) class III phosphatidylinositol 3-kinase core complex is a positive regulator^11^. The serine/threonine protein kinase AMP-activated protein kinase (AMPK) also positively regulates autophagy.

AMPK consists of three subunits: a catalytic *α*-subunit and two regulatory *β*- and γ-subunits, each of which is present in different isoforms (*α*1, *α*2, *β*1, *β*2, γ1, γ2, γ3). Phosphorylation of the *α*-subunit at Thr172 by upstream kinases in the activation loop is a prerequisite for AMPK activity^12^. AMPK directly regulates autophagy by phosphorylating and thereby activating ULK1. Four phosphorylation sites within ULK1 (Ser467, Ser555, Thr574 and Ser637) have been mapped. These optimally match the AMPK substrate motif, which is conserved in higher eukaryotes^13^. Ser757 within ULK1 (ULK1-Ser757) is also phosphorylated by mTOR^14^. Although we have observed the alterations of ULK1 and p-ULK1 in prion infected cell models, the exact situations of AMPK and AMPK-ULK1 pathway in brain tissues of prion diseases remain unknown.

In our previously study, we showed that the macroautophagic system is activated in scrapie-infected experimental animals and in certain subtypes of human prion diseases, in which the essential negative regulator mTOR is severely inhibited^15^. More recently, we found that FBXW7 (F-box and WD repeat domain containing 7) is responsible for the induction of mTOR degradation and for the ability of endogenous autophagy to counteract prion replication in the scrapie infected cell line SMB-S15^16^. As one of the important elements in autophagy induction, both ULK1 and ULK1-Ser555 are increased in hamsters infected with scrapie agent-263 K and in SMB-S15 cells. Since ULK1-Ser555 is a target of AMPK, alterations in the former suggest that multi-kinase cross-talk and protein turnover pathways participate in the regulation of autophagy during prion infection.

In this study, we analysed AMPK and ULK1 in the brains of hamsters infected with scrapie agent-263 K and in the scrapie infected cell line SMB-S15. Our results showed that autophagic flux was activated in SMB-S15 cells. AMPK, AMPK-Thr172, ULK1 and ULK1-Ser555 were markedly up-regulated in both experiment models. Additionally, LKB1, which is associated with AMPK activation, was selectively increased in the brains of the scrapie infected hamsters during the early and middle stages of infection. In SMB-S15 cells, the knockdown of endogenous ULK1 not only depressed ULK1-Ser555, but also inhibited LC3 lipidation. Thus, together with the abolishment of the mTOR regulatory pathway, activation of the AMPK-ULK1 pathway contributes to autophagy activation in prion-infected brain tissues.

## Materials and Methods

### Reagents and antibodies

Rabbit anti-AMPK*α*1/2 (sc-25792) was purchased from Santa Cruz. Rabbit anti-ULK1 (#5723-1) was purchased from Epitomics. Rabbit anti-phospho-AMPK*α* (Thr172) (#2535), rabbit anti-phospho-ULK1 (Ser555, #5869) (Ser757, #6888), rabbit anti-CaMKII (#4436), rabbit anti-BECN1 (#3738) and anti-mTOR (#2972) were purchased from Cell Signaling Technology. Rabbit anti-LKB1 (L7917) and rabbit anti-p62/SQSTM1 (P0067) were purchased from Sigma. Rabbit anti-LC3 (NB100-2220) was obtained from Novus Biologicals. Mouse anti-*β*-actin (Sr-25113) was purchased from Subrray Biotechnology. A mouse PrP-specific antibody 6D11 (sc-58581) that recognises PrP^C^ and PrP^Sc^ was purchased from Santa Cruz. Horseradish peroxidase (HRP)-conjugated goat anti-mouse or rabbit IgG (#31430 or #31460) was purchased from Thermo. Both si-RNA-ULK1 (Stealth RNAi) and si-RNA-NC were designed and synthesised by Life Technologies. DAPI (D1306) was from Invitrogen. Lysosome inhibitor NH_4_Cl (A9434) was purchased from Sigma. Protease inhibitor cocktail set III was from Merck (539134) and the enhanced chemoluminescence system (ECL) was from PerkinElmer (NEL103E001EA). Lipofectamine 2000 was purchased from Life Technologies.

### Animal and human brain samples

In this study, infection was carried out by intracerebrally inoculating the hamsters with 2 *μ*l of 10% brain homogenates infected with scrapie agent 263 K^17^. The normal controls were injected with an equal amount of physical saline. The brains were surgically removed from the euthanised animals at the terminal stage of infection (about 80 dpi at their last gasp), and then either frozen at −80 °C or fixed in 10% buffered formalin. Additionally, brain samples of euthanised 263 K-infected hamsters were collected 0, 10, 30, 50 and 70 days post-inoculation (dpi). Brain homogenates from three infected hamsters were collected and pooled at each of the above time-points. The use of animal specimens in this study was approved by the Ethical Committee of the National Institute for Viral Disease Prevention and Control, China CDC, under protocol 2009ZX10004-101. Animal housing and experimental protocols were in accordance with the Chinese Regulations for the Administration of Affairs Concerning Experimental Animals.

### Cell culture and cell lysates

SMB-PS and SMB-S15 cells derived from mice neurons were obtained from the Roslin Institute (UK) and maintained in medium 199 containing Earle’s salts and supplemented with 5% newborn calf serum and 10% fetal calf serum^18^. SMB-S15 cells were infected with scrapie strain Chandler; PrP^Sc^ replication was maintained by cell passage. SMB-PS cell are SMB-S15 cells that were completely cured of the prion by pentosan sulfate (PS) treatment; they are without detectable PrP^Sc18^. Cultured cells were washed twice with cold PBS and harvested with cold lysis buffer (100 mM NaCl, 10 mM EDTA, 10 mM Tris, 0.5% sodium deoxycholate, 0.5% Nonidet P-40, pH 7.5) containing a mixture of protease inhibitors from cocktail set III. The lysate was placed on the ice for 30 min and then centrifuged at 10,000 *g* at 4 °C for 15 min. The supernatants were collected and stored at −20 °C until needed.

### Cell transfection

SMB-S15 cells were plated in 6-well plates for 24 h before transfection. Transfection of siRNA-ULK1 (sense: 5′-GGGUAGUAAUGACACCACCUCGGAA-3′; antisense: 5′-UUCCGAGGUGGUGUCAUUACUACCC-3′) was conducted using 75 pmol of RNA oligo per well with Lipofectamine 2000 according to the manufacturer’s instructions. At 24 h post-transfection, the cells were harvested for use in further experiments.

### Preparation of brain tissue samples

Brain homogenates from normal and scrapie-infected hamsters were prepared according to a protocol described previously^19^. Briefly, the hamster brains were washed three times with ice-cold TBS (10 mM Tris-HCl, 133 mM NaCl, pH 7.4) and homogenised in lysis buffer (100 mM NaCl, 10 mM EDTA, 0.5% Nonidet P-40, 0.5% sodium deoxycholate in 10 mM Tris-HCl, pH 7.4) containing a mixture of protease inhibitors cocktail set III. The homogenates were centrifuged at 2000 *g* for 10 min and the supernatants were stored at −80 °C until needed.

### Western blot

Aliquots of brain homogenates and cell extracts were separated on 8% or 10% acrylamide gels by SDS-PAGE and electroblotted onto a nitrocellulose membrane using a semi-dry blotting system (Bio-Rad). The membranes were blocked with 5% (w/v) bovine serum albumin (BSA) in 1 ×  Tris-buffered saline containing 0.1% Tween 20 (TBST) at room temperature (RT) for 2 h and then probed individually with the primary antibodies at 4 °C overnight. Blots washed with TBST were incubated with 1:5000 HRP-conjugated goat anti-mouse or rabbit IgG at RT for 2 h and then developed using the ECL system. The bands were visualised on autoradiography films and photographed using the ChemiDoc XRS^+^ Imager (Bio-Rad).

### Immunohistochemistry (IHC) assays

The hamster brains were fixed in 10% formalin and embedded in paraffin. For IHC, paraffin slides (5-μm thick) were deparaffinised in xylene and ethanol. Antigen retrieval was performed insodiumcitratebuffer (pH 6.0) by heating for 30 min followed by enzyme digestion for 1 min. The slides were quenched in 3% hydrogen peroxidefor 15 min andpermeabilised in 0.3% Triton X-100 for 20 min. The sections were blocked with 1% normal goat serum at RT for 15 min and then incubated at 4 °C overnight with 1:50-diluted polyclonal antibody (pAb) for AMPK, 1:50-diluted monoclonal antibody (mAb) for phospho-AMPK*α* (Thr172), 1:100-diluted pAb for ULK1 and phospho-ULK1 (Ser757), 1:100-diluted anit-ULK1 mAb and anti-ULK1-Ser555. The slides were then incubated with 1:250-diluted HRP-conjugated goat anti-rabbit secondary antibody at 37 °C for 1 h and visualised by incubation with 3,3′-diaminobenzidine tetrahydrochloride (DAB). The slices were counterstained with hematoxylin, dehydrated and mounted in Permount.

### Immunofluorescence assay (IFA)

SMB cells were washed with PBS and fixed with 4% formaldehyde for 20 min at RT, followed by permeabilisation with 0.3% Triton in PBS for 15 min. The cells were then blocked in 5% normal goat serum at RT for 30 min and incubated at 4 °C overnight with primary antibodies. After three washes with PBS, the cells were incubated with secondary antibodies at RT for 1 h, washed again and stained with DAPI for 20 min. The slices were mounted with Permount and viewed using an Olympus FV1000 microscope.

### Statistical analysis

All of the experiments were conducted at least three times, with consistent results. Immunoblot images were quantitatively analysed using software Image J. Statistical analysis was performed using GraphPad Prism software. Significance was determined using a *t* test. A P value < 0.05 was considered to to be statistically significant. All data values are presented as the mean ± SD.

## Results

### AMPKα1/2 in the brains of hamsters with terminal-stage scrapie 263 K infection

**Figure 1:**
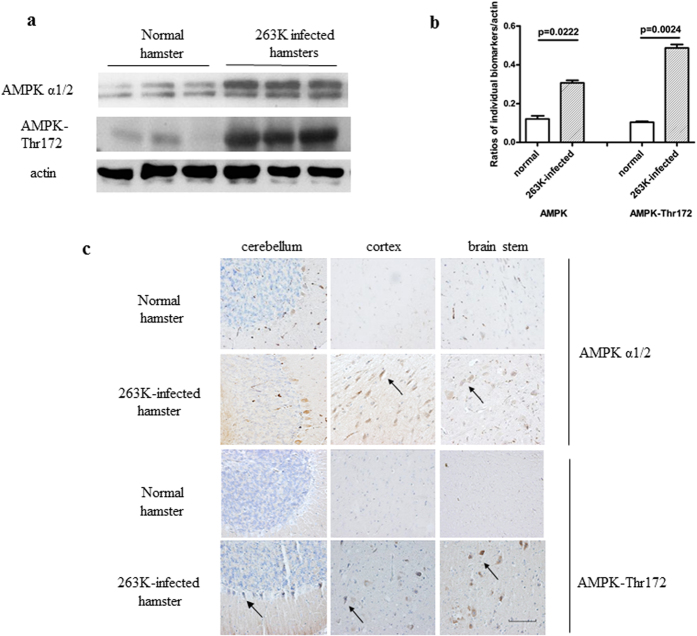
AMPK and AMPK-Thr172 expression in the brains of hamsters at the terminal stages of infection with scrapie agent 263 K. (**a**) Western blots. (**b**) The results of the quantitative analysis of the gray values of AMPK in infected and normal animals after normalisation of the values with *β*-actin. Graphical data denote the mean ± SD (n = 4). All of the gels were run under the experimental conditions detailed in the Methods section. Full-length blots were cropped for final display. (**c**) Representative immunohistochemistry (IHC) assays of AMPK and AMPK-Thr172 in the cortex, cerebellum and brainstem of 263 K-infected hamsters and in the normal control (×40). Scale bar = 100 μm.

To observe potential changes in the endogenous kinase AMPKα1/2 and its phosphorylated form AMPK-Thr172 in prion-diseased brain tissue, 10% brain homogenates from four hamsters infected with scrapie strain 263 K were evaluated. Western blots showed that the levels of AMPK*α*1/2 (p = 0.0222) and AMPK-Thr172 (p = 0.0024) were significantly higher in infected than in normal animals (Fig. 1a,b). Further standardization of the ratio of the average values of AMPK-Thr172 to the total amount of AMPKα1/2 showed an increased tendency of a higher ratio in the brains of the infected hamsters, albeit without statistical difference compared with the ratio in the normal control (Supplemental Fig. 1a). Alterations in AMPK were also examined by IHC using slides prepared from the brains of normal and 263 K-infected hamsters. AMPKα1/2 and AMPK-Thr172 positivity was higher in the cerebellum, cortex and brainstem of 263 K-infected animals than in the same brain regions of normal animals (Fig. 1c). These results suggested a trend towards the up-regulation of AMPK in scrapie-infected animals.

### Alterations of ULK1 and ULK1-Ser555 in the brains of hamsters with terminal-stage scrapie 263 K infection

**Figure 2:**
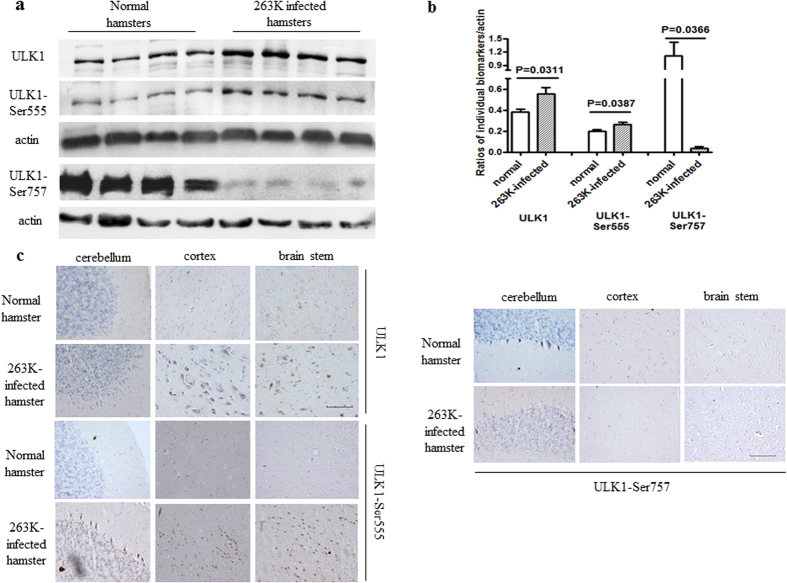
ULK1 and phospho-ULK1 expression in the brains of hamsters at the terminal stage of infection with scrapie agent 263 K . (**a**) Western blots. ULK1, ULK1-Ser555 and ULK1-Ser757 are shown on the left. (**b**) Results of a quantitative analysis of the gray values of ULK1 in infected and normal animals after normalisation with *β*-actin. Graphical data denote the mean ± SD (n = 4). All of the gels were run under the experimental conditions detailed in the Methods section. Full-length blots were cropped for final display. Representative IHC assays of ULK1 (**c**) and (d) ULK1-Ser555 and ULK1-Ser757 in the cortex, cerebellum and brainstem of 263 K-infected hamsters and normal control (×40). Scale bar = 100 μm.

To determine whether brain ULK1 is altered in prion disease, its levels were compared in 10% brain homogenates from normal and 263 K-infected hamsters. Western blots showed significantly higher brain ULK1 levels in 263 K-infected hamsters than in normal controls (p = 0.0311) (Fig. 2a,b). Quantitative analysis of western blots showed a significant (p = 0.0387) increase in the expression of ULK1-Ser555 and a significant (p = 0.0366) decrease in the expression of ULK1-Ser757 in the brain homogenates of 263 K-infected vs. normal hamsters (Fig. 2a,b). In addition, the ratios of the amounts of each of the two phosphoproteins to the total amount of ULK1 showed a significant decrease in ULK1-Ser757 (P = 0.0487) in the 263 K-infected brains compared with normal hamsters whereas the ratio of ULK1-Ser555 was the same in the two groups (Supplemental Fig. 1b). Thus, in infected brain tissues the fraction of ULK1-Ser555 increased in parallel with ULK1, while the fraction of ULK1-Ser757 was significantly reduced. IHC assays for ULK1 and its phosphorylated isoforms in brain sections prepared from 263 K-infected and normal hamsters showed strongly positive signals of ULK1 and ULK1-Ser555 in the cerebellum, cortex and brainstem of scrapie-infected animals (Fig. 2c,d). Compared with the normal controls, ULK1-Ser757 signals in the cerebellum of 263 K-infected hamsters were clearly weaker, while those in cortex and brain stem were similar (Fig. 2d).

### Enhanced autophagic flux in the scrapie-infected cell line SMB-S15

**Figure 3:**
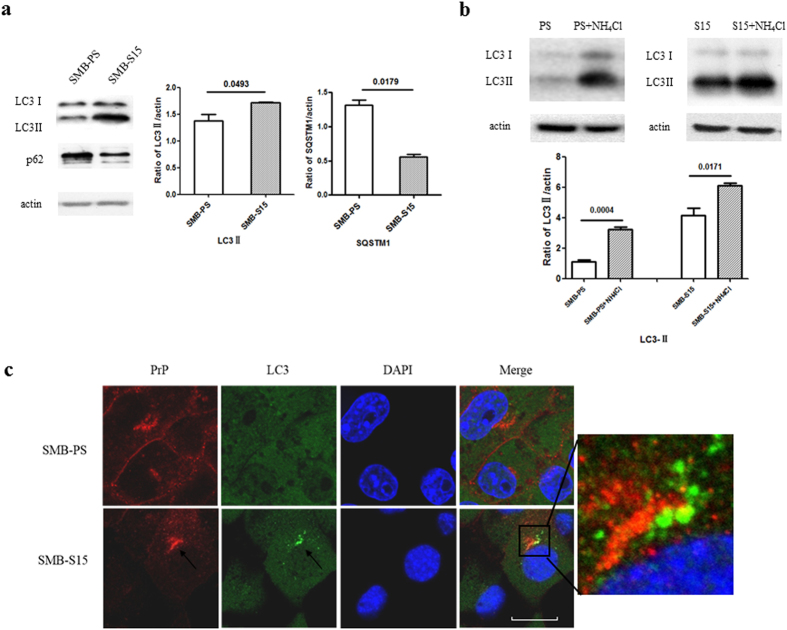
Autophagy activity in the scrapie-infected cell line SMB-S15. (**a**) Western blots of LC3 and SQSTM1 in SMB cells. LC3-I, LC3-II and SQSTM1 are shown on the left. The results of the quantitative analysis of the gray values of the LC3-II and SQSTM1 blots in SMB-S15 cells and control SMB-PS cells are shown on the right. The average relative gray value was calculated from three independent blots and expressed as the mean ± SD. All of the gels were run under the experimental conditions detailed in the Methods section. Full-length blots were cropped for final display. (**b**) Western blot of LC3 in SMB cells in the presence or absence of NH_4_Cl. The average relative gray value was calculated from three independent blots and expressed as the mean ± SD. (**c**) Representative confocal microscopy images of SMB cells double-stained for LC3 and PrP. The PrP (red), LC3 (green), DAPI (blue) and merged images are indicated on the top. The magnification view of merged graph of SMB-S15 cells is shown on the left. Scale bar = 20 μm.

Autophagy in cultured cells that support prion replication *in vitro* was studied in cell line SMB-S15, infected with scrapie strain Chandler. LC3-II signals were detected in the lysates of SMB-S15 cells but were barely present in SMB-PS cells (p = 0.0493), while SQSTM1 signals were significantly weaker in SMB-S15 cells than in SMB-PS cells (p = 0.0179) (Fig. 3a). To determine whether autophagosome accumulation was due to autophagy induction or to suppression of the late maturation and degradation stage of autophagy, SMB cells were exposed to the lysosomal inhibitor NH_4_Cl and analysed by western blot, which showed that LC3-II levels were enhanced in the presence of NH_4_Cl (Fig. 3b). In SMB-15 cells double-stained with PrP- and LC3-specific antibodies and analysed in an IFA, brilliant green LC3 particles were seen in the cytoplasm of SMB-S15 but not of SMB-PS cells. Meanwhile, PrP signals (red) did not colocalise but were juxtaposed with LC3 signals (green) (Fig. 3c). In SMB-PS cells treated with the autophagy activator rapamycin, immunofluorescence LC3 puncta accumulated in the cytoplasm (Supplemental Fig. 2). Those results indicated an enhanced autophagic flux in SMB-S15 cells and were in line with our previous findings in a study of the brains of 263 K-infected hamsters^15^.

### Less decreased mTOR in SMB cells than that in the brains of scrapie infected hamsters

**Figure 4:**
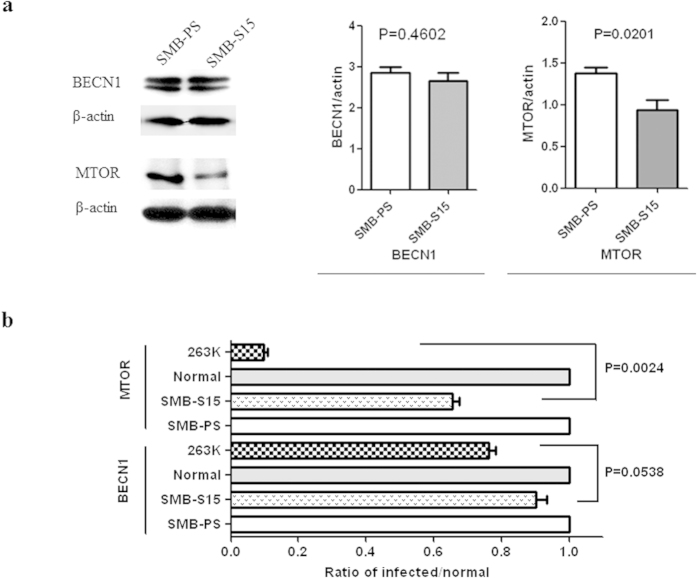
Evaluation of BECN1 and mTOR in SMB cells. (**a**) Western blots of BECN1 and mTOR. The results of the quantitative analysis of the gray values of the individual blots of SMB-S15 and SMB-PS cells after normalisation with *β*-actin are shown on the right. The average relative gray value was calculated from three independent blots and expressed as the mean ± SD. (**b**) Comparison of the alterations in BECN1 and mTOR in SMB-S15 cells and in the brains of hamsters infected with scrapie agent 263 K. Data on the infection of the hamsters were from our previous publication^15^. The alterations of BECN1 and mTOR in SMB-S15 cells and in 263 K-infected hamsters were assessed relative to those of SMB-PS cells (set at 1.0) and normal hamsters (set at 1.0), respectively. Statistical differences in the alterations of BECN1 and mTOR between the scrapie-infected cell line (SMB-S15) and scrapie-infected animals (263 K-infected hamsters) are shown on the right.

In our previous study, the large reductions in mTOR in prion-infected brain tissue greatly enhanced the activation of autophagy^15^. To determine whether similar alterations in autophagy regulatory elements occur in SMB cells, the levels of BECN1 and mTOR were evaluated quantitatively on western blots. As shown in Fig. 4a, BECN1 signals were the same in SMB-S15 as in SMB-PS cells whereas mTOR signals were weaker mTOR (p = 0.0201). To determine whether the degree of mTOR reduction following prion infection differed *in vitro* and *in vivo*, the relative mTOR gray values of mTOR and BECN1 were analysed in the western blots of SMB cells in this study and in those from our previous study of the brain tissues of 263 K-infected and normal hamsters^15^. The values were calculated separately after their normalisation with respect to *β*-actin. As shown in Fig. 4B, the level of BECN1 in SMB-S15 cells was reduced by ∼90% compared with the control SMB-PS cells, while in 263 K-infected hamsters it was reduced to 70% of that in normal hamsters; the difference was not statistically significant (p = 0.0538). By contrast, the level of mTOR in SMB-S15 cells was ∼65% of that in SMB-PS cells, while mTOR in 263 K-infected hamsters it was ∼10% of the level in normal hamsters; the difference was significant (p = 0.0024).

### Alterations of AMPK and ULK1 in scrapie-infected SMB-S15 cells

**Figure 5:**
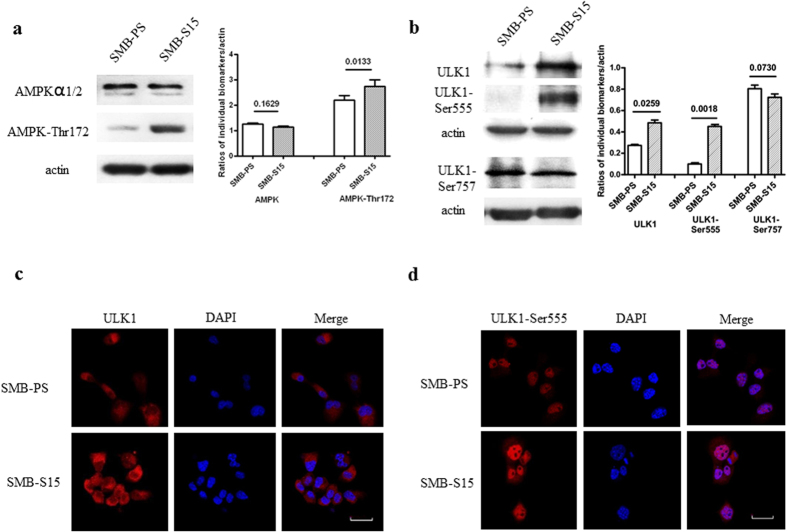
Evaluation of AMPK and ULK1, as well as their phospho-isoforms in SMB cells. (**a**) Western blots of AMPK and AMPK-Thr172. The results of the quantitative analysis of the gray values of the individual blots. The average relative gray value was calculated from three independent blots and expressed as the mean ± SD. (**b**) Western blots of ULK1, ULK1-Ser555 and ULK1-Ser757. Quantitative analysis of the gray values of the individual blots. All of the gels were run under the same experimental conditions, as detailed in the Methods section. Full-length blots were cropped for final display. (**c**) Representative confocal microscopy images of ULK1 in SMB-S15 and SMB-PS cells. The ULK1 (red), DAPI (blue) and merged images are indicated above. (**d**) Representative confocal microscopy images of ULK1-Ser555 in SMB-S15 and SMB-PS cells. The ULK1-Ser555 (red), DAPI (blue) and merged images are indicated above. Scale bar = 20 μm.

To determine the phosphorylation status of AMPK and ULK1 in scrapie-infected cells *in vitro*, lysates of SMB-S15 and SMB-PS cells were analysed by western blotting. Compared with normal SMB-PS cells, AMPKα1/2 levels in scrapie-infected SMB-S15 cells were essentially unchanged (p = 0.1629) whereas there was a remarkable increase in AMPK-Thr172 (p = 0.0133) (Fig. 5a). The level of ULK1 in SMB-S15 cells was markedly up-regulated (p = 0.0259), paralleling an increase in ULK1-Ser555 (p = 0.0018) and a decrease in ULK1-Ser757 (p = 0.0730) (Fig. 5b). In addition, compared with SMB-PS cells, in SMB-S15 cells the ratios of AMPK-Thr172/AMPK*α*1/2 (p = 0.0061) and ULK1-Ser555/ULK1 were significantly higher (p = 0.0251) whereas the ratio of ULK1-Ser757/ULK1 was significantly lower (p = 0.0044) (Supplemental Fig. 3). It seems that the activation profiles of AMPK and ULK1 in the prion infected cells *in vitro* share similarities with that of the prion infected animals *in vivo*.

To verify the above observations, cultured SMB cells were immunofluorescently stained with antibodies against ULK1 and ULK1-Ser555. The expression of these two proteins differed in western blots of the two cell lines. Confocal microscopy showed relatively more intensive ULK1-Ser555 signals in SMB-S15 cells than in SMB-PS cells (Fig. 5c,d).

### Reduced expression of upstream proteins of AMPK in the brains of hamsters with terminal-stage scrapie 263 K infection and increased expression in scrapie-infected SMB-S15 cells

**Figure 6:**
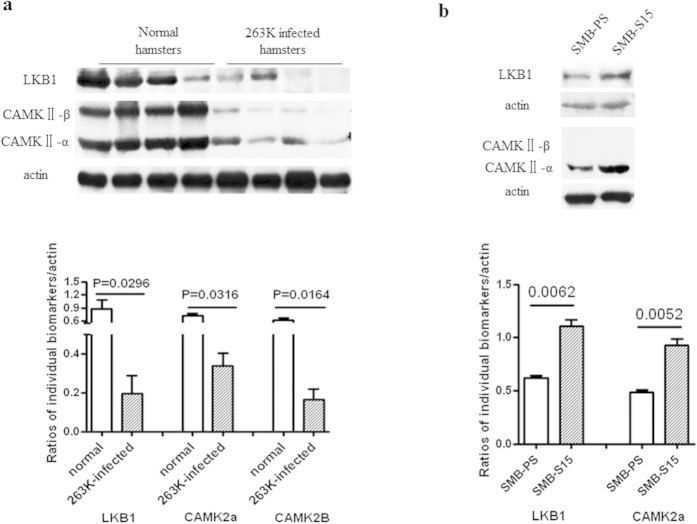
Evaluation of LKB1 and CAMKII in the brain tissues of 263 K-infected hamsters (**a**) and in the scrapie-infected cell line SMB-S15 (**b**). The results of the quantitative analysis of the gray values of the individual blots from the scrapie-infected group and the normal control are presented below each graph. For the animal samples, the graphical data are presented as the mean ± SD (n = 4). For the cell samples, the means ± SDs of the data from three independent blots are shown. All of the gels were run under the same experimental conditions, as detailed in the Methods section. Full-length blots were cropped for final display.

The expression of LKB1 and CAMKII (CAMKII-α and CAMKII-β), as upstream regulators of AMPK, in the brain tissues of scrapie-infected hamsters and cell lines was investigated by western blotting. In the brain tissues of 263 K-infected hamsters, the levels of both LKB1 (p = 0.0296) and CAMKII (CAMKII-*α* p = 0.0316 and CAMKII-β p = 0.0164) were markedly lower than in the normal controls (Fig. 6a). However, in SMB-S15 cells, the levels of LKB1 (p = 0.0062) and CAMKII-*α* (p = 0.0052) were higher than in control SMB-PS cells (Fig. 6b). CAMKII-*β* was not detected in either of the SMB cell lines. These results implied a different functional state of AMPK regulators between actively proliferating prion-infected cells *in vitro* and terminal prion-infected brain tissues *in vivo*.

### Dynamic alterations of AMPK and ULK1 in scrapie-infected hamsters

**Figure 7:**
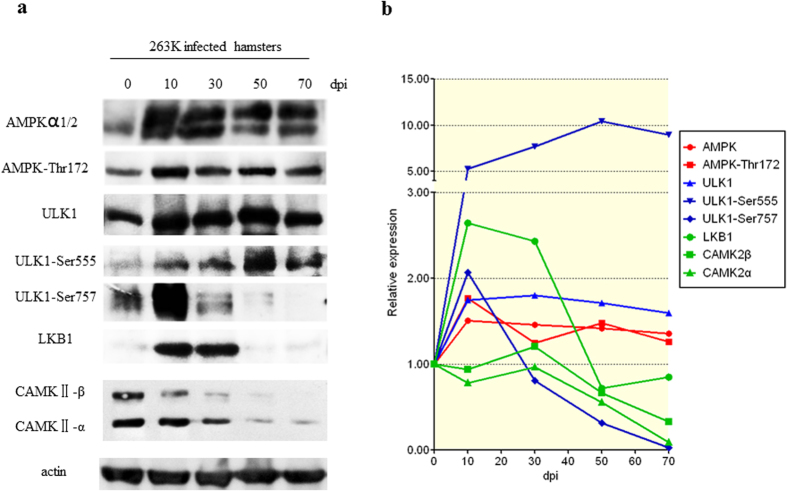
Dynamic assays of AMPK, ULK1 and their activators in the brain tissues of 263 K-infected hamsters during the 70-day incubation period. The brain samples were collected 0, 10, 30, 50 and 70 days post-infection (dpi). Brain homogenates from three individual hamsters collected at each time-point were pooled. (**a**) Western blots of AMPK, AMPK-Thr172, ULK1, ULK1-Ser555, ULK1-Ser757, LKB1 and CAMKII. The same amount of brain homogenate was loaded in each lane for SDS-PAGE. (**b**) The results of the quantitative analysis of each gray value from the individual blots relative to the *β*-actin value. The expression of each marker was normalised to that at 0 dpi. All of the gels were run under the same experimental conditions, as detailed in the Methods section. Full-length blots were cropped for final display.

To follow the changes in AMPK and ULK1 during scrapie infection of hamster brain tissues, the brains of 263 K-infected hamsters were collected at 10, 30, 50 and 70 days post-inoculation (dpi), pooling the replicate samples from each time point. As shown in Fig. 7, in the serial samples of 263 K-infected hamsters (mean incubation time: 66.7 ± 1.1 days), AMPK*α*1/2 and AMPK-Thr172 increased rapidly during the early stage of infection and were maintained at high levels until the end of the experiment. ULK1 also increased during the early stage. ULK1-Ser555 levels rose beginning at 10 dpi and reached a peak at 50 dpi, while following an increase in ULK-Ser757 during the early stage, it decreased rapidly beginning at 30 dpi and almost disappeared by 70 dpi. LKB1 was up-regulated at 10 and 30 dpi but was subsequently down-regulated. CAMKII-*α* and -*β* remained unchanged until 70 dpi, at which time the levels of both forms were lower. Those data illustrate that a constantly changed situation of AMPK, ULK1 and their related factors during prion infection.

### Knockdown of ULK1 decreases LC3 II levels in scrapie-infected SMB-S15 cells

**Figure 8:**
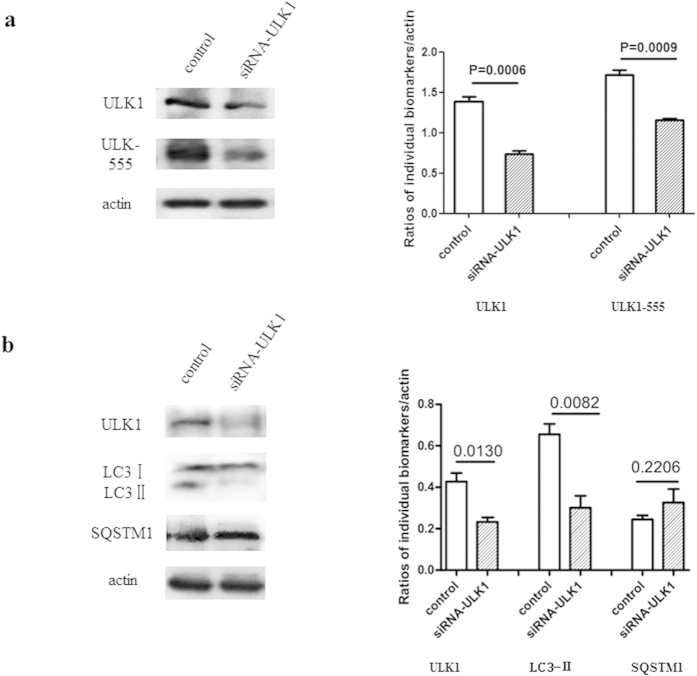
Changes in the indicators of autophagy after knockdown of ULK1 in scrapie-infected SMB-S15 cells. Western blots of (**a**) ULK1 and ULK1-Ser555 and (**b**) LC3-II and SQSTM1. SMB-S15 cells were transiently transfected with siRNA-ULK1. The results of the quantitative analyses of ULK1, ULK1-Ser555, LC3-II and SQSTM1 signals relative to *β*-actin are shown. The means ± SDs of data from three independent blots are shown. All of the gels were run under the same experimental conditions, as detailed in the Methods section. Full-length blots were cropped for final display.

To gain insight into the regulation of autophagy by ULK1 in prion-infected cells, SMB-S15 cells were transiently transfected with the ULK1-specific siRNA. Western blots showed that in these cells ULK1 levels were ∼45% of those in the negative control cells (Fig. 8a). In cells transfected with the ULK1-specific siRNA, ULK1-Ser555 was markedly weaker than in control cells (p = 0.0009). Along with the knockdown of cellular ULK1 and ULK1-Ser555, significant reductions in LC3-II (p = 0.0082) were determined quantitatively (Fig. 8b). However, the knockdown of ULK1 did not result in significant changes in the cellular level of SQSTM1 (p = 0.2206, Fig. 8b). Thus, the knockdown of ULK1 in cultured prion-infected cells efficiently inhibited LC3 lipidation.

## Discussion

Autophagy is an important host cellular mechanism to eliminate excess or damaged proteins, protein complexes and organelles. In animals experimentally infected with scrapie and in certain subtypes of human prion diseases autophagy is up-regulated^15^. Several signalling pathways are involved in the regulation of autophagy^20^,^21^,^22^,^23^,^24^, including mTOR, a strongly negative regulator of autophagy in healthy animals. In hamsters with terminal-stage scrapie agent 263 K infections, mTOR expression is strongly diminished whereas the activity of class III PI3K, a positive regulator of autophagy, is maintained^15^. In addition to these two pathways, AMPK activates autophagy by phosphorylating and activating ULK1. Conversely, the phosphorylation of ULK1 by mTOR inhibits its activity^4^, which prevents its interaction with AMPK^14^,^25^,^26^,^27^,^28^. AMPK was shown to phosphorylate the tuberous sclerosis complex (TSC1/2), which inhibits mTOR pathways^29^.

In this study, we showed an up-regulated trend of AMPK in the brains of hamsters infected with the scrapie agent 263 K beginning at the very early stage of infection and maintained for as long as 70 dpi. In parallel with the increase in brain AMPK, brain levels of ULK1 and especially of its phosphorylated form ULK1-Ser555 increase over the course of a scrapie infection. Since phosphorylation of the conserved Thr172, located in the activation loop of the catalytic *α*-subunit of AMPK, is a prerequisite for AMPK activity^30^, the levels of AMPK-Thr172 were significantly higher in the prion-infected cell line SMB-S15 than in its normal counterpart SMB-PS. It is reasonable to speculate that the activity of AMPK was increased during prion infection *in vitro*. In line with the increased activity of AMPK, the levels of ULK1 and ULK1-Ser555 were significantly higher in SMB-S15 cells than in SMB-PS cells. Thus, the AMPK-ULK1 pathway is activated by prion infection *in vitro.* In addition to abolishing the interaction of mTOR, the activated AMPK-ULK1 pathway may also contribute to enhancing autophagic flux during prion infection.

Coincidental with the decrease of mTOR in hamster brains infected with scrapie 263 K, the level of ULK1-Ser757, the downstream substrate of mTOR, increased transiently at the very early stage of infection but then quickly declined and was barely detectable at 70 dpi. Phosphorylation of ULK1 at Ser757 disrupts the association between AMPK and ULK1^31^. In the brain tissues of the scrapie-infected hamsters, down-regulation of ULK1-Ser757 during the middle and late stages of infection reflected an activated AMPK-ULK1 pathway. In SMB-S15 cells, however, there was little change in ULK1-Ser757 despite a marked reduction in the ratio of ULK1-Ser757/ULK1. This difference in ULK1-Ser757 between prion infection *in vitro* and *in vivo* correlated well with mTOR levels under the two conditions. The weaker reduction in mTOR in SMB-S15 cells may be related to the relatively stable level of ULK1-Ser757 during prion replication *in vitro.* In addition to Ser555 and Ser757, other sites of ULK1 phosphorylation have been described, such as Ser467, Thr574 and Ser637^13^,^32^,^33^,^34^,^35^. Whether those sites are also altered during prion infection remains to be determined. Also unknown is whether, as in skeletal muscle, activated AMPK inhibits mTORC1^36^ in the brains of animals with prion disease.

LKB1, a tumour suppressor serine-threonine kinase also referred to as STK11^37^, is expressed in the brain tissues of mammals^38^. CAMKII is a calcium/calmodulin-dependent kinase II that plays a fundamental role in learning and memory^39^,^40^. Both are positive regulators of AMPK via phosphorylation at Thr172^41^. LKB1 expression was strongly increased in the brains of 263 K-infected hamsters during the early stage of infection but decrease to the normal level during the mid- and late-stages, which suggests that AMPK is up-regulated by LKB1 immediately after scrapie infection of animal tissues. CAMKII-*α* and -*β* levels were fairly stable in the brain tissues of 263 K-infected hamsters throughout the incubation period. However, along with the extensive neuronal degeneration at the terminal stage, CAMKII expression was reduced to extremely low levels. In contrast to the dynamic changes in LKB1 and CAMKII-*α in vivo*, in SMB-S15 cells the levels of both proteins remained relatively high during cell replication *in vitro*. We recently demonstrated that activated autophagic flux in SMB-S15 cells involves the clearance of PrP^Sc16^. Thus, the maintenance of elevated levels of LKB1 and CAMKII in prion-infected cells may help to reduce PrP^Sc^ replication to an acceptable level via autophagy.

AMPK is expressed in various metabolism-associated organs and acts as a sensor of the overall cellular energy level. As such, it regulates energy metabolism to maintain cellular homeostasis. When nutrient is sufficient, Mg^2+^-ATP promotes the dephosphorylation of AMPK, resulting in its inactivation, while insulin and growth factors activate TORC1 through upstream kinases including AKT1 (protein kinase B) and extracellular signal regulated kinase (MAPK3-MAPK1 or ERK). In response to energy depletion, such as glucose starvation, TORC1 is inhibited by AMPK and autophagy is stimulated^42^. Our previous analyses of global gene expression profiles in brain tissues of humans with genetic TSEs showed that almost half of the affected cellular pathways are metabolism-associated, such as amino acid metabolism, energy metabolism and lipid metabolism^43^,^44^. Neuronal defects in glucose uptake and metabolism following reductions in the glucose transporter 3 (GLUT3) are commonly observed in various scrapie-infected rodent models and in the scrapie-infected cell line SMB-S15^45^. Those data strongly highlight a close linkage between activation of the AMPK-ULK1 pathway and energy preservation in brain tissues during prion infection.

Although the AMPK-ULK1 pathway was similarly altered during prion infection *in vivo* and *in vitro*, differences were also observed, especially in brain samples collected at the terminal stage of infection. Thus, except for AMPK, ULK1 and ULK1-Ser555, the levels of the proteins examined in this study were almost undetectable in terminally infected brains whereas they were maintained in the prion-infected cultured cells. In prion-infected hamsters, prion replication and infection inevitably cause irreversible brain damage and death; during this process, the levels of many neuronal proteins decrease or disappear, along with the extensive loss of neuron. This conclusion is supported by similar changes at the transcriptional levels in the postmortem brain tissues of patients with fatal familial insomnia, another prion disease. However, in prion-infected cell cultures, prion replication does not cause cell death, such that proteins involved in the cell cycle and responsible for cell growth and viability are preserved. In fact, alterations in the AMPK-ULK1 pathway and the activation of autophagy were in some ways comparable in the prion-infected cell line and scrapie-infected brains collected at the early and middle stages of infection. However, in prion-infected brains, activated autophagy cannot compete with the fatal neurotoxicity of prion infection and may even aggravate neuronal damage through autophagic cell death (ACD) or type II programmed cell death^46^,^47^,^48^,^49^, whereas in the cultured cells it may help the host cells to balance prion replication and cell duplication.

In summary, this study presented evidence of an alteration in the AMPK-ULK1 pathway during prion infection, both *in vitro* and *in vivo*, that contributes to the activation of autophagy. As important biological factors in many different tissues of mammals, AMPK and ULK1 have multiple functions. This study focussed on their regulation of autophagy. The participation of other biological processes in the pathogenesis of prion infection and prion disease remains to be elucidated.

## Additional Information

**How to cite this article**: Fan, X.-Y. *et al.* Activation of the AMPK-ULK1 pathway plays an important role in autophagy during prion infection. *Sci. Rep.* **5**, 14728; doi: 10.1038/srep14728 (2015).

## Supplementary Material

Supplementary Information
