# Supplementary material for: Activation of the AMPK-ULK1 pathway plays an important role in autophagy during prion infection
Source: Sci Rep. 2015 Oct 1;5:14728. doi: 10.1038/srep14728 (PMC4589734; doi:10.1038/srep14728)
Supplement: Supplementary Information [file srep14728-s1.pdf]

Supplementary information for

Activation of the AMPK-ULK1 pathway plays an important role in autophagy during prion infection

Xue-Yu Fan[1,2*], Chan Tian[2*], Hui Wang[1,2*],Yin Xu[2*], Ke Ren[2], Bao-Yun Zhang[2], Chen Gao[2], Qi Shi[2], Ge Meng[2], Lu-Bin Zhang[1], Yang-Jing Zhao[1], Qi-Xiang Shao[1], Xiao-Ping Dong[2.3]

[1] Department of Immunology, and Jiangsu Key Laboratory of Medical Science and Laboratory Medicine, School of Medicine, Jiangsu University, Zhenjiang 212013, Jiangsu, China

[2] State Key Laboratory for Infectious Disease Prevention and Control, Collaborative Innovation Center for Diagnosis and Treatment of Infectious Diseases (Zhejiang University, Hangzhou 310003), National Institute for Viral Disease Control and Prevention, Chinese Center for Disease Control and Prevention, Chang-Bai Rd 155, Beijing 102206, China

[3] Chinese Academy of Sciences Key Laboratory of Pathogenic Microbiology and Immunology, Institute of Microbiology, Chinese Academy of Sciences, Beijing 100101, China

*These authors contributed equally to this article.

Corresponding authors: Prof. Xiao-Ping Dong, Fax: +86-10-58900815. Email: dongxp238@sina.com; Prof. Qi-Xiang Shao, Email: shao_qx@ujs.edu.cn

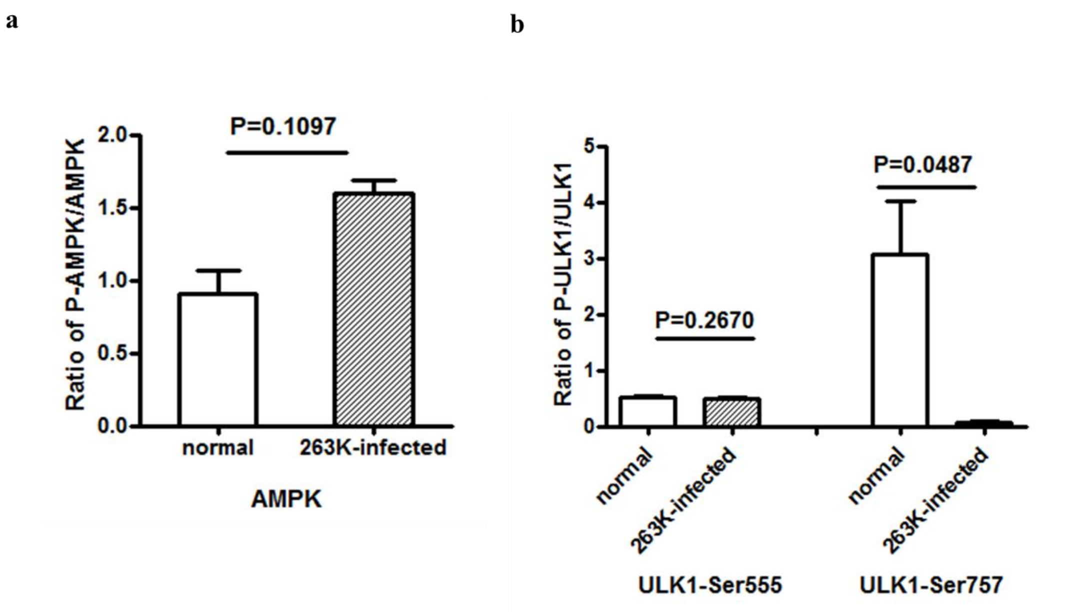

Supplemental Figure 1 Quantitative analyses of the ratios of p-AMPK/AMPK and p-ULK1/ULK1 in the brain tissues of 263K-infected and normal hamsters. (a) Ratio of p-AMPK/AMPK. (b) Ratios of ULK1-Ser555/ULK1 and ULK1-Ser757/ULK1.

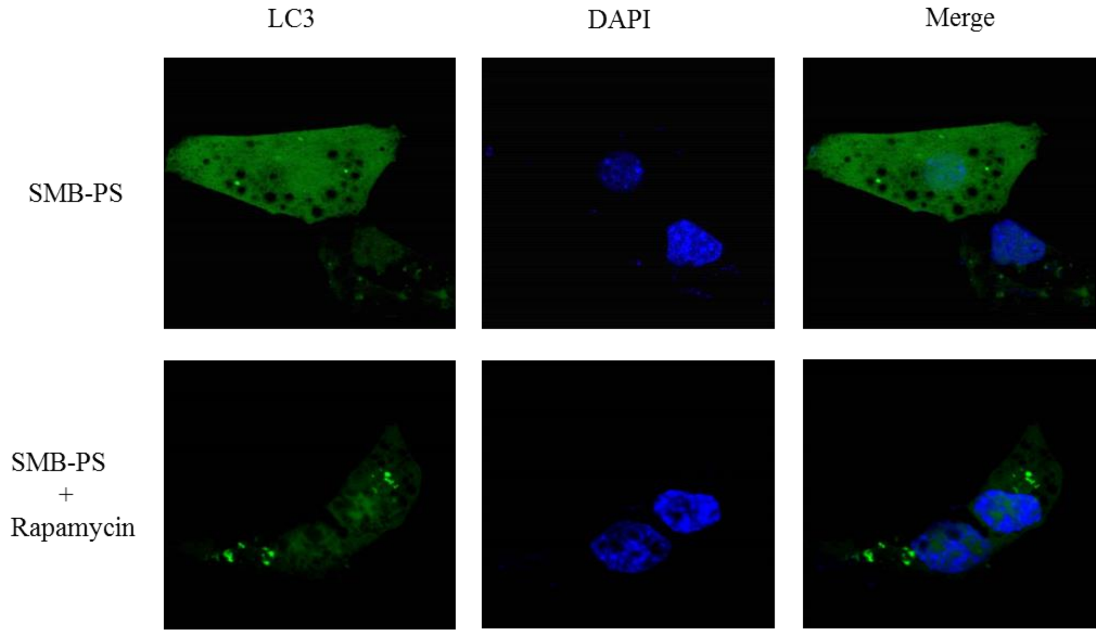

Supplemental Figure 2 Representative confocal microscopy images of SMB-PS cells immunofluorescently stained for LC3 treated with (lower panel) or without (upper panel) rapamycin. The images of LC3 (green), DAPI (blue) and merge are indicated on the lower panel. Scale bar = 20 μm.

a b

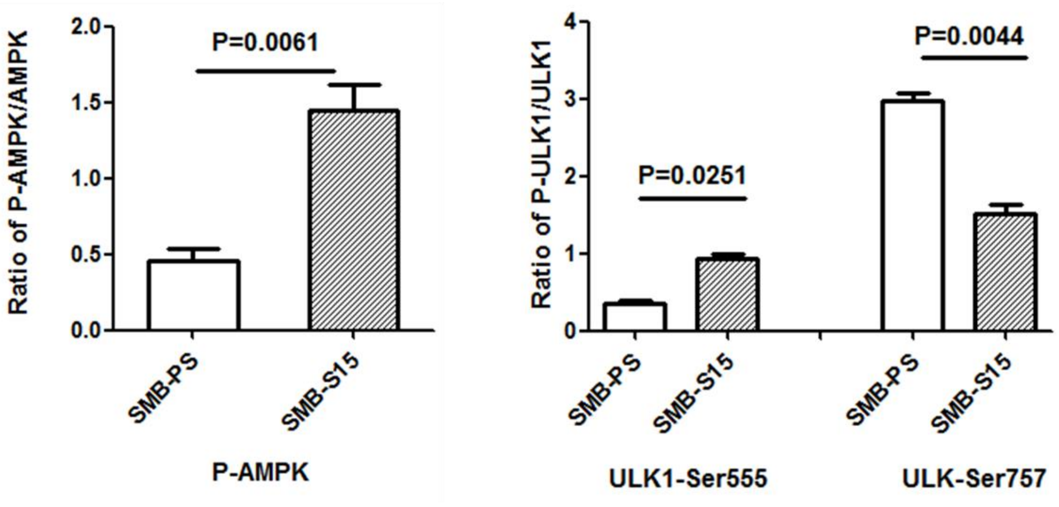

Supplemental Figure 3 Quantitative analyses of the ratios of p-AMPK/AMPK and p-ULK1/ULK1 in SMB-S15 and SMB-PS cells. A. Ratio of p-AMPK/AMPK. B. Ratios of ULK1-Ser555/ULK1 and ULK1-Ser757/ULK1.
